# HIA and EIA Are Different, but Maybe Not in the Way We Thought They Were: A Bibliometric Analysis

**DOI:** 10.3390/ijerph18179101

**Published:** 2021-08-28

**Authors:** Jinhee Kim, Fiona Anne Haigh

**Affiliations:** 1 Centre for Health Equity Research Training and Evaluation (CHETRE), Australia Research Centre for Primary Health Care & Equity, A Unit of Population Health, Member of the Ingham Institute, The University of New South Wales, Sydney, NSW 2052, Australia; jinhee.kim@unsw.edu.au; 2 Centre for Primary Health Care & Equity, A Unit of Clinical Services Integration and Population Health, Health Equity Research Development Unit (HERDU), The University of New South Wales, Sydney Local Health District, Sydney, NSW 2052, Australia

**Keywords:** health impact assessment, environmental impact assessment, paradigm, bibliometric analysis, public health

## Abstract

Background: The fields of Health Impact Assessment (HIA) and Environmental Impact Assessment (EIA) have grown with increasing numbers of disciplines and sectors contributing to their advancements, but with it, perceived conflict over methodological and disciplinary approaches to integrate health in impact assessments. This study maps the current field of HIA and health in EIA to examine the scientific landscape of the field. Methods: We carried out a bibliometric analysis of HIA papers and EIA papers that included a health focus in peer-reviewed journals in the Web of Science Core Collection (*n* = 229). We carried out co-authorship and co-citation network analyses of authors and documents in VOSviewer. Results: We identified two main co-authorship and co-citation groupings. Our document co-citation analysis also identified four clusters with two major groups, the Defining HIA cluster and the Describing the fields cluster versus the Active transport quantitative HIA cluster, and the Quantitative modelling tools cluster. Conclusion: Our findings strongly suggest that there exist two groups of thought in the scholarly fields of HIA and health in EIA. Barriers to developing more methodologically integrated approaches to considering health within EIA are related more to disciplinary differences than field (HIA versus EIA)-based differences and we advocate for the development of transdisciplinary approaches to both HIA and EIA.

## 1. Introduction

Recent legislative developments such as the European Union Environmental Impact Assessment (EIA) Directive (2011/92/EU amended by 2014/52/EU) requiring the consideration of health within EIAs have strengthening the need to integrate methodological and disciplinary approaches. Cave et al., in this Special Issue, argue that “Health in EIA requires a framework that applies to topics with different methodological and epistemological frameworks. A single assessment will present numerous findings on different topics and can, therefore, use both quantitative and qualitative approaches” [1] (p. 11).

There is now a body of literature on the relationship between a health impact assessment (HIA) and EIA and how health is considered within EIA [1,2,3,4]. Reviews carried out analysing how health is considered within EIA tend to emphasise a narrow biomedical interpretation of health within EIA with a focus on environmental determinants of health [3]. This contrasts with HIA literature that tends to emphasize a broader conceptualisation of health [5,6].

Implicit (and sometimes explicit) within the literature are differences between disciplinary and paradigm perspectives—a so called paradigm war where paradigm and related epistemological and methodological differences are seen as incommensurate [7]. Paradigms embody views that are held in relation to ontology (What is out there to know?), epistemology (What and how can we know about it?), methodology (How can we go about acquiring knowledge?), methods (What procedures can we use to acquire it?), and axiology (values of being, about what human states are to be valued simply because of what they are) [8,9,10].

Both HIA and EIA are concerned with creating knowledge about the potential impacts of an activity (be it a policy, project, or programme). We (and others) believe that the dominant disciplines within EIA and HIA fields tend to have different paradigm perspectives [4,5,11,12]. EIA is largely positivist in epistemological origin [5] and draws strongly on fields of biological sciences, epidemiology, toxicology, risk assessment, cost–benefit analysis, ecology, and increasingly sociological disciplines [12]. HIA, when it emerged as a field in the 1990s, had two main drivers; EIA and Healthy Public Policy [13]. In contrast to EIA’s positivist disciplinary tendencies, healthy public policy draws more from the fields of social sciences, political economy, and political science and tends to be more socially constructivist in epistemological origin [5,13,14,15].

Generally, fields that have tended to utilise a positivist or post positivist paradigm share a view that what is knowable is what can be observed and measured. These fields privilege certain methodologies and the methods used within research, with a greater focus on quantitative approaches (counting things) relying on empirical observation and measurement [16]. Whereas fields that draw more on social constructivist or interpretivist paradigms where reality is constructed from “interaction between human beings and their world” [17] (p. 42), draw more on qualitative research analysing text rather than numbers and focusing more on understanding meaning [18].

The reality is more complex and nuanced than presented here, with individual practitioners and researchers within the different fields having a mix of paradigm perspectives and not all quantitatively inclined researchers being positivists and qualitative, social constructivists. However, for the purpose of this paper, our focus is on identifying overarching tendencies and characteristics within the fields of HIA and EIA and our main point is that fields and the dominant disciplines within them tend to draw on particular paradigm positions and these paradigm positions tend to be associated with certain types of methodological approaches. This influences the questions asked, the methodological approaches used, and the value placed on different types of data.

These differing paradigms are often not made explicit or, in some cases, practitioners may not even be aware of them. However, as Carter and Little [19] observed, it is impossible to create knowledge “without at least tacit assumptions about what knowledge is and how it is constructed” and we see the impact of these paradigm perspectives in disagreements and conflicts between the fields but also in assessments themselves [12].

In this paper we utilise bibliometric analysis techniques to investigate the broad fields of ‘health impact assessment’ and ‘health in environmental impact assessment’. The following questions guided our analysis:

1. Are there any distinct clusters of literature and authors within the fields of HIA and health in EIA literature?
2. If there are clusters:
  (a) What is the nature of linkages between clusters?
  (b) Can we identify distinct paradigm and/or disciplinary boundaries between clusters?
  (c) Can we identify key themes and areas of focus within clusters?

## 2. Methods

### 2.1. Data Collection

To collect the publications for the bibliometric analysis, we searched for articles in peer-reviewed journals in the Web of Science Core Collection of which the main topic of study was EIA with an explicit health component or HIAs. The search terms “health impact assessment*” OR “HIA” OR “HIAs” were applied in the title or author keyword fields to capture articles addressing health impact assessments and the search terms “health” AND (“environmental impact assessment*” OR “EIA” OR “EIAs”) were applied to capture publications that address health in the context of environmental impact assessments and strategic environmental impact assessments. Additional limiters included language (English), years (2015–2020), and type of publication (article). Early access publications were excluded. Although our bibliometric analysis includes all the cited references that were listed in the reference list of the publications as the data source, we included only the years 2015–2020 to analyse the co-citation pattern in the current scientific literature.

We used the Gothenburg definition of HIA:

A combination of procedures, methods, and tools by which a policy, program, or project may be judged as to its potential effects on the health of a population, and the distribution of its effects within the population [20];

The IAIA definition of EIA:

refers to a formal and systematic process that includes identifying, predicting, evaluating, and mitigating the biophysical, social, and other relevant effects of development proposals prior to major decisions being taken and commitments made [21].

We purposely used the Web of Science as our sole bibliographic database because the visualisation software tool we used, VOSviewer, has maximum functionality for Web of Science and does not support mixed use of other databases. Limiting the search to Web of Science did not introduce any critical limitations to the study because Web of Science is a comprehensive scientific database, and we could assume saturation was achieved. Additionally, this was fit for purpose because the aim of the study was to examine the overall pattern of the two fields and not a comprehensive statistical analysis.

The search retrieved a total of 556 items. We then imported the search results to Covidence to exclude irrelevant studies that did not address HIA as ‘procedures, methods and tools by which a policy, programme or project may be judged as to its potential effects on the health of a population, and the distribution of those effects within the population’ [20]. We included papers that were full HIA or ‘health in EIA’ case studies that prospectively or concurrently addressed health impacts of proposals, partial reports, reviews of methodologies, or conceptual papers. The final number of publications included for the bibliometric analysis was 229. The metadata and the list of references were retrieved and exported from Web of Science and imported into VOSviewer for bibliometric analysis.

### 2.2. Bibliometric Analysis

Visualising bibliometric networks provide a powerful and efficient method to understanding the overall landscape of the topic area by quantifying and illustrating the relationships of publications. Several software tools such as Pajek, Gephi, VOSViewer, CiteSpace, Sci^2^, Cytoscape, CitNetExplorer, HistCite have been developed for analysing and visualising bibliometric and citation networks [22]. In our analysis, we used VOSviewer which offers an easy-to-use software tool that has a strong focus on visualisation. VOSviewer is especially equipped for visualising larger networks and offers some text mining features.

There are many different types of bibliometric mapping and analytical methods that can be performed of authors, journals, publications, countries, and institutions and can be applied at levels of titles, keywords, text, and even entire citation records [23,24]. In this paper, we conducted co-authorship and co-citation network analyses of authors and documents.

Co-authorship relationships show scientific collaboration patterns of authors and visualise the existing research communities in the field [22,23,24]. When two authors co-author a publication, a co-authorship relationship is established. The more publications two authors co-author, the stronger the co-authorship relationship. Groups of authors who co-author frequently appear as distinct clusters in the co-authorship network.

Co-citation relationships, on the other hand, visualise the intellectual structure of the scientific field [22,23,24]. Two publications are co-citated if there is a third publication that cites both publications. To be strongly co-cited, more publications must cite the two earlier works. Frequently, co-cited papers indicate that they contain the key concepts, methods, or experiments in the field that have received peer recognition and the co-citation patterns can be used to map out the relationships between these key ideas. Therefore, we could interpret that strong co-citation links indicate subject similarity and the co-occurrence of ideas. Clusters of a close co-citation relationship between documents can be seen as belonging to the same ‘research front’. The validity of co-citation analysis as a tool for understanding the intellectual structure of a scientific discipline has been demonstrated through numerous studies [25,26,27,28]. Co-citation network mapping can be performed at the document, author, or journal levels.

Because co-citation relationships are based on the cited documents, external publications were added to the co-citation analysis. Furthermore, because co-citation is a relationship which is established by the citing authors, co-citation patterns can change over time. Changes in the co-citation patterns, when viewed over a period of years, may provide clues to understanding the mechanism of specialty development, or changes in the paradigm. In this paper, the co-citation map was produced on articles published during the years 2015–2020, which shows the current intellectual structure of the HIA and health in EIA fields.

## 3. Results

The findings are presented in two parts—we first identified two major groups of co-authorship clusters, which was further supported by an author co-citation analysis. To further confirm the pattern, we looked at the document level and found four distinct co-citation clusters. A content analysis of the top co-cited papers in each cluster is presented. Throughout the paper, we used the term clusters to describe the distinctive aggregation of closely related publications or authors that were represented in different colours. The term group represents closely related clusters.

### 3.1. Co-Authorship Network

**Figure 1:**
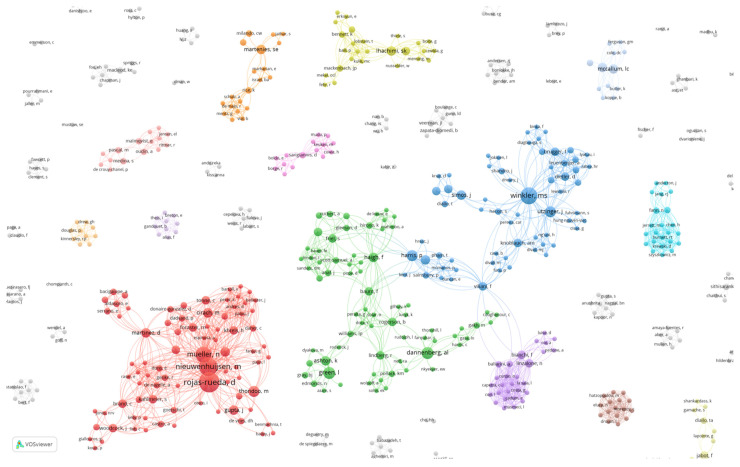
Co-authorship map of 229 publications on health in EIA and HIA. Each circle denotes an author. The size of the circles indicates the total link strength of the author. The larger the circle, the stronger the total strength of the co-authorship links of the author with other authors. In other words, we can assume that the authors with a larger circle are nodes in the clusters. The distance between circles represents the relative strength of the co-author relationships. The colours represent clusters of close co-authorship relationships.

In the network of the 229 publications included in the analysis, there were 880 authors. The co-authorship network of these authors is depicted in Figure 1. The co-authorship map shows that there exist 11 distinct clusters with at least 15 authors who co-author publications frequently. There appeared to be two major co-authorship groups. The largest cluster depicted in red (Rojas-Rueda et al. cluster) was the most prolific in the field. The second group consists of the next largest clusters, shown in the order of green (Green et al. cluster), blue (Winkler et al. cluster), yellow (Lhachimi et al. cluster), and purple (Bianchi et al. cluster). It is worth noting that the largest cluster (Rojas-Rueda et al. cluster) was not connected with the other larger clusters.

A closer examination of the authors and their disciplinary backgrounds (based on job title and publicly available information on academic qualifications) (Table 1) showed that the authors in the largest cluster (Rojas-Rueda et al. cluster) were all linked to the Barcelona Institute of Global Health and were predominantly from the environmental epidemiological disciplinary background. The authors in the interconnected group represented a wider range of disciplinary backgrounds, including public health, epidemiology and environmental health, and social and political sciences. Publications in the Rojas-Rueda et al. cluster tended to have an environmental health focus with a particular interest in (active) transport and health. There were also methodological publications focussing mainly on quantitative approaches to assessment. The authors in the Green et al. cluster tended to focus on the purpose, functions, effectiveness, and methods of HIAs and included reviews and case studies focused on areas of application. Authors in the Winkler et al. cluster focused on current global practice, the role of HIA in EIA, as well as HIA case studies. The Lhachimi et al. cluster focussed particularly on the application of one quantitative modelling approach, Dynamo HIA.

### 3.2. Author Co-Citation Network

**Figure 2:**
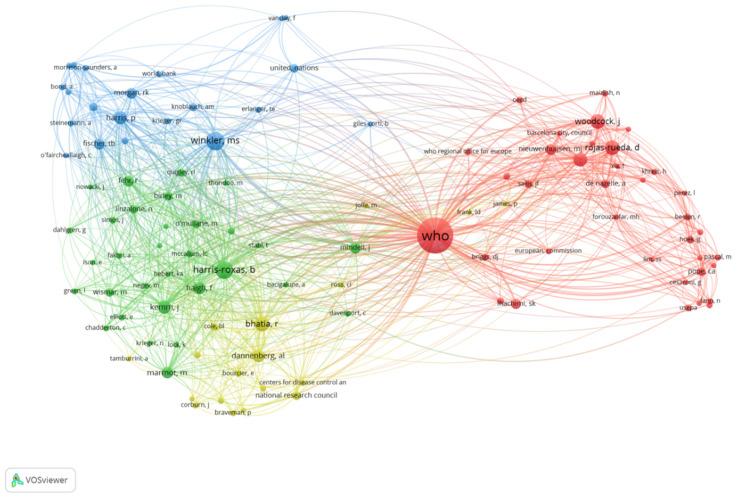
Co-citation network of authors. Each circle represents an author. The size of the circles denotes the number of citations; the larger the circle, the more times the author was cited. The distance between the circle indicates the relative strength of the co-citation relationship. The thickness of the lines represents the frequency of co-citation relationships.

The co-authorship pattern was consistent with the author co-citation network (Figure 2). The author co-citation network showed four clusters of co-citation relationships. The largest cluster (red) was distinctly separated from the other three clusters suggesting there were two categories of research traditions in the field. Moreover, the authors in the largest co-authorship cluster (in red) in Figure 1 could also be found in the largest author co-citation cluster (in red) in Figure 2. The authors in the inter-connected group in Figure 1 (green and blue) were consistent with the key authors in the three closely related clusters on the left side in Figure 2 (green and blue). These findings strongly suggest that there exist two groups of thought in the scholarly fields of HIA and health in EIA.

### 3.3. Document Co-Citation Network

**Figure 3:**
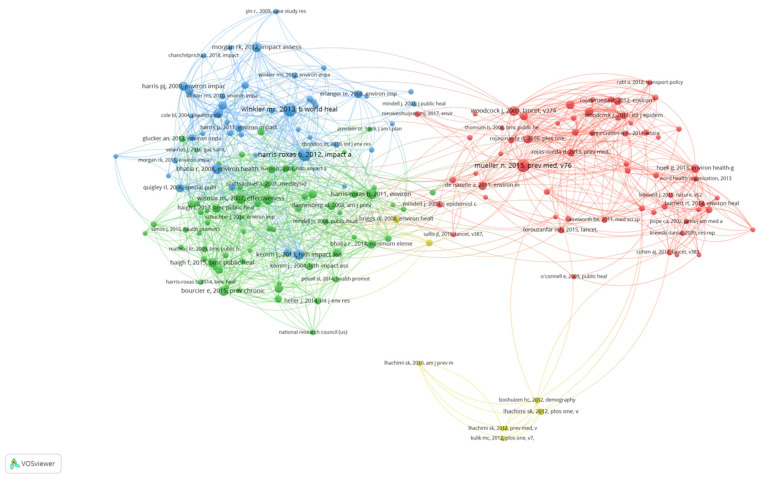
Document co-citation map of 229 publications on health in EIA and HIA.

The document co-citation network showed four clusters of strong co-citation relationships (Figure 3). Documents belonging to the same cluster were frequently co-cited by many publications, suggesting subject similarity and the co-occurrence of ideas. There appeared to be two major groups, the Defining HIA cluster (Green) and the Describing the fields cluster (Blue) versus the Active transport quantitative HIA cluster (Red), and the Quantitative modelling tools cluster (Yellow). From a closer examination of the highly co-cited publications from each cluster as listed in Table 2, we could make the following interpretations.

It was difficult to distinguish the differences between the Defining HIA cluster (Green) and the Describing the fields cluster (Blue). It did seem to suggest that the Defining HIA cluster (Green) addressed HIA while the Describing the fields cluster (Blue) contained discussions about both HIA and Health in EIA. In the Defining HIA cluster (Green), there were two main types of papers: 1. large scale evaluations of HIA effectiveness and 2. papers defining and describing HIA. An examination of the disciplinary perspective of the (lead) authors identified an overarching public health disciplinary perspective with a tendency towards disciplines with an implicit social constructivist or interpretivist paradigm perspective. Meanwhile, the Describing the fields cluster (Blue) differentiated itself from the Defining HIA cluster (Green) in that it focussed on papers describing the field of HIA, Health in EIA and EIA, with the authors making recommendations to strengthen the fields. This cluster demonstrates a mix of disciplinary perspectives covering public health/medicine, environmental epidemiology, and social research with a tendency more towards positivist paradigm perspectives than the Defining HIA cluster (Green).

The Active transport quantitative HIA cluster (Red) and the Quantitative modelling tools cluster (Yellow) tended to show characteristics of the positivist methodological aspects of HIAs or EIAs such as epidemiological evidence and quantification and modelling for the assessment of health impacts. The Active transport quantitative HIA cluster (Red) focussed on the relationship between the active transport and health with papers containing modelling and/or estimating health impacts of scenarios utilizing quantitative assessment methods. In comparison to the Defining HIA cluster (Green) and the Describing the fields cluster (Blue), the main disciplinary perspective was epidemiology with a clear positivist paradigm perspective. The Quantitative modelling tools cluster (Yellow) also shared a strong positivist paradigm perspective with environmental epidemiology being the dominant discipline. These papers either described quantitative HIA tools or their application.

We also noted that, when we were screening papers for inclusion in the study, a number of papers with a strong quantitative modelling methodological focus used the term HIA differently than we as HIA practitioners and academics would. In the field of HIA, there is a very strong procedural element involving screening, scoping, identifications, assessment, and the development of recommendations [5,13]. In our set, there were a number of papers that identified as HIA papers but did not report carrying out HIA steps such as screening, scoping, or developing recommendations. These papers also did not tend to identify common HIA procedural and administrative aspects such as the formation of steering groups and decision-making processes. These HIAs were more closely aligned procedurally with an environmental risk assessment.

When examining the geographic focus of the papers, we could see that the Defining HIA cluster (Green) and the Describing the fields cluster (Blue) had an international spread of papers, whereas the more technical Active transport quantitative HIA cluster (Red) and the Quantitative modelling tools cluster (Yellow) were predominantly European in focus.

When examining the co-citation map (Figure 3), it was also clear that the Defining HIA cluster (Green) and the Describing the fields cluster (Blue) were closely linked, with a slot of co-citation between documents in both groups, whereas there was very limited cross-over between the Defining HIA cluster (Green) and the Describing the fields cluster (Blue) and the Active transport quantitative HIA cluster (Red) and no links with the Quantitative modelling tools cluster (Yellow). This suggests that that the more positivist/quantitatively focussed clusters (Active transport quantitative HIA cluster (Red) and the Quantitative modelling tools cluster (Yellow) were not drawing on the literature from the more procedurally focussed clusters (Defining HIA cluster (Green) and Describing the fields cluster (Blue)) or vice versa—they were in sense operating in parallel.

Each circle denotes a cited publication, and the lines indicate co-citation relationships. The size of the circle represents the number of citations. The larger the circle, the more frequently the publication was cited. The colours indicate clusters of publications with strong co-citation relationships.

## 4. Discussion

Our findings showed the current overall landscape of the field of Health in EIA and HIA and two major groups of research traditions (or paradigms) within the fields could be identified. The two research traditions were not defined by differences of the field, (EIA versus HIA), but by disciplinary, methodological, or paradigmatic differences.

The bibliometric networks showed a clear cluster of quantitative, environmental health focused publications and authors (shown in red) sitting separately from the other main clusters (green and blue) in terms of co-citation and co-authorship. This does not necessarily mean that the authors and papers were not engaging with each in other ways, but this did not show up in the bibliometric analysis. Interestingly, although the focus of the papers was environmental health, they were within the context of HIA. We were unable to tell from our analysis whether this was linked to environmental determinants being more amenable to quantification than social determinants or whether the field and discipline interest drove the focus on environmental health pathways. Environmental health determinants are of particular relevance to health within EIA projects, given the material physical nature of those projects; however, we also saw these quantitative methodologies being applied within the HIA papers. These methodological differences could be linked to implicit paradigm differences, with quantitative methods being more strongly linked to positivist paradigm perspectives and qualitative or mixed methods being linked to social constructivist/interpretivist paradigm positions.

Furthermore, and more importantly, our analysis showed that there was crossover between HIA and Health in EIA in terms of co-authorship and co-citation, with some authors acting as boundary spanners bringing together the fields of HIA and EIA (e.g., Baum and Villiani). Where we did see separation was in the discipline, methodology, and paradigm. The two groups that appeared in the document co-citation clusters demonstrated this with public health and mixed methods focused as a distinctive group, and quantitative methods focused and environmental and social epidemiology as the other. The fields of HIA and health in EIA were not separate; rather, they were represented in both groups. In conclusion, these findings suggested that barriers to developing more methodologically integrated approaches to considering health within EIA were related more to disciplinary, methodological, and paradigmatic differences than field (HIA versus EIA)-based differences. Or, in other words, it’s not so much that HIA and EIA are not integrated but rather that the core disciplines (with their paradigm and methodological differences) within the fields are not integrated. This suggests that if we wanted to strengthen the consideration of health in impact assessments through a greater methodological integration, we should focus on ways of bringing the actors from different disciplines who work within HIA and EIA together.

We, the authors of this paper, both drew on the critical realist paradigm to inform our work. From our paradigm perspective, it was not an either-or question to answer when determining what methodological approach is best suited to assess health impacts within an EIA. From a critical realist perspective, our environment is layered, ranging from the sub molecular to planetary level and there is interplay and emergence between those layers [46]. This means that different methodological approaches and data may be required to describe the casual pathways and the interplay between them. This requires drawing on a range of disciplines and applying methodologies based on what can answer the research questions rather than disciplinary preferences.

The value of HIAs is maximized by bringing together concepts, methodologies, and solutions from diverse backgrounds and disciplines. In doing so, a transdisciplinary approach that addresses both the overlapping and non-overlapping areas of the disciplines is required. This differs from multidisciplinary or interdisciplinary approaches as these approaches seek collaboration in the intersections of consensus or avoid areas that are in conflict [47]. Articulating the contradictions and the commonalities, as we have demonstrated in this study, facilitates transdisciplinary approaches to impact assessment research and practice as it enables researchers to make sense of the knowledge in the wider context and understand the apparent conflicts between disciplinary knowledge.

A bibliometric analysis is a powerful tool that visualises the overall landscape of a scientific field and presents a descriptive overview of the relationships of the selected publications in an efficient way. However, caution is required in the interpretation and replication of the results. Results from bibliometric analyses vary depending on the search and inclusion criteria and the clustering parameters. The parameters are selected based on the research question which, in this case, was influenced by the researchers’ underlying beliefs and paradigms in relation to HIA. For example, both researchers in this review are HIA practitioners and researchers and both hold a critical realist paradigm position that influences our understanding of the determinants of health and the function of HIA as a tool in the policymaking process. As such, we drew on a broad social model of health, participatory methods, and mixed methods. The positions of the researchers not only prompted the overall research question but were reflected in the execution of the bibliometric review. Firstly, in screening the publications, we applied a set of inclusion criteria that would include HIAs that addressed the broader social concept of health and a wider range of methodologies applied in assessing health impacts. A search, filtering, and analysis by reviewers who follow positivist paradigm or come from biomedical fields may produce different results and interpretation than reviewers who embrace constructivist or the social model of health paradigms by setting a different set of inclusion criteria. Secondly, in determining the patterns of clusters, what we identify and interpret as unique clusters may be different to what others may find as clusters of significance.

Some limitation should be considered in understanding the findings of our review. The publications were searched from one multidisciplinary database, a decision determined due to technical reasons. While searching from only one database may be criticized for a systematic literature review that emphasizes comprehensive and exhaustive searches, this method was adequate as the database is one of the largest multidisciplinary and comprehensive academic databases. We limited our search to English language publications but were aware of significant French and Spanish and other localized non-English fields of work that we did not include. We limited our search to papers that were identified as either HIA or EIA. We did not explicitly search for other forms of impact assessment that may include health such as the Integrated Environmental Health Impact assessment, or the Strategic Health Impact Assessment or Social Impact Assessment. Future research could consider health in all forms of impact assessment; however, this was beyond the scope of our paper. In our analysis of disciplinary backgrounds of authors within clusters, we based our assumptions on the job title and publicly available information of degrees held. This may not align with how the authors identify their disciplinary perspective. We included peer-reviewed journal publications from the recent 5 years as we believe they will be sufficient to demonstrate the current landscape of the field of HIA. Future work would look at other time frames to examine how the field has changed over time. Despite the limitations of bibliometric analyses, this methodology is fit-for-purpose.

Our study was the first bibliometric analysis carried out in the fields of HIA and EIA. By providing an overview of the current leading clusters of publications and authors our paper provides an introduction and synopsis of the current state of play in academic literature. Future research could build on our work through, for example, examining how the field has developed and changed over time and also considering how different clusters assess health impacts. We would also welcome and encourage researchers with differing disciplinary perspectives to engage with and offer alternative interpretations of our data.

## 5. Conclusions

A bibliometric analysis of the fields of HIA and Health in EIA demonstrated that there are existing linkages between the fields of HIA and EIA. In a sense, the fields are already talking to each other. However, there were also groupings of authors and their publications that sat in isolation from the broader fields.

We argue that understanding the full range of potential health impacts requires more than multiple disciplines working in parallel. In a health and environmental impact assessment, multiple causal mechanisms operate at multiple levels. This requires multi-disciplinarity to understand different levels, inter-disciplinarity to understand multi causality and emergence and transdisciplinary work that goes beyond disciplines working in parallel or sequence to utilise integrative approaches [48,49]. From our perspective, strengthening transdisciplinary approaches in the impact assessment is a necessary task in order to be able to adequately identify and act on health impacts.

In terms of next steps, we call on our colleagues to work with us to find a way to bring the different clusters and disciplinary actors together in a conversation where we can learn from each other’s perspectives and identify ways to collaborate and engage in transdisciplinary work. For effective transdisciplinary collaboration to occur, it is important that researchers and practitioners understand the epistemological and ontological differences that underpin the knowledge and ideas that are generated. Furthermore, researchers and practitioners should actively apply transdisciplinary knowledge to draw a more coherent and comprehensive approach to HIA. By doing so, we will be able to attempt collaboration in the non-overlapping or marginal areas between the diverse disciplines rather than limiting collaboration to those overlapping areas. In doing so, as O’Campo reminds us “the ultimate goal of transdisciplinary approaches is to achieve social change by generating knowledge about real-world problems and solutions” [50].

## Figures and Tables

**Table 1:** Main co-authorship clusters of 229 publications on health in EIA and HIA.

Cluster	Key Authors	Main Focus of Publications	Disciplinary Perspective
Rojas-Rueda et al. cluster (depicted in red in Figure 1)	Rojas-Rueda, D.; Nieuwenhuijsen, M.; Mueller, N.; Thondoo, M.; Cirach, M.; Gascon, M.	Transport (cycling, active transport), air pollution, greenspace/ tree canopy, health impact assessment (quantitative methods), integrated impact assessment, HIA methods (participatory quantitative), urban health HiAP, HIA application/legislation	Environmental epidemiology, medical anthropology, public health
Green et al. cluster (depicted in green in Figure 1)	Green, L.; Dannenberg, A.; Haigh, F.; Ashton, K.	HIA, role of HIA, HIA methods Effectiveness of HIA, HIA case studies and reviews (agriculture, food, nutrition, criminal justice, energy and natural resources, infrastructure, trade)	Public health, epidemiology, environmental health, law, social research
Winkler et al. cluster (depicted in blue in Figure 1)	Winkler, M.; Utzinger, J.; Harris, P.; Riley, E.	HIA application/current practice (Latin America, Sub-Saharan Africa, Vietnam, global), HIA and sustainable development, role of HIA Health in EIA, HIA case study (trade)	Epidemiology, environmental sciences, public health, political science, urban planner
Lhachimi et al. cluster (depicted in yellow in Figure 1)	Lhachimi, S.; Boshuizen, H; Schoenbach, J.; Mackenbach, J.	HIA quantitative methods application, quantitative HIA case study (processed meat, saturated fat)	Statistician, health economics, demography, public policy, political science

**Table 2:** Top five publications with the highest total link strength in each document co-citation cluster.

**Cluster 1 (Red)—Active Transport Quantitative HIA**		
Mainly active transport and health-focused papers modelling and/or estimating health impacts of scenarios utilizing quantitative assessment methods. Main disciplinary perspective epidemiology with an implicit positivist paradigm perspective.		
	(Mueller et al., 2015) *Health impact assessment of active transportation: A systematic review* [29]	Systematic review of studies conducting HIA of a mode shift to active transport. Quantitative risk assessment focused HIA model. Discipline—environmental epidemiology. Europe, United States, Australia, and New Zealand.
	(Woodcock et al., 2009) *Public health benefits of strategies to reduce greenhouse-gas emissions: urban land transport* [30]	Comparative Risk Assessment to estimate and model the health effects of alternative urban land transport scenarios for two scenarios. Discipline—epidemiology. UK, India.
	(Woodcock et al., 2014) *Health effects of the London bicycle sharing system: health impact modelling study* [31]	Study modelling the health impacts of a bicycle share system. Discipline—epidemiology. UK.
	(Hoek et al., 2013) *Long-term air pollution exposure and cardio- respiratory mortality: a review* [32]	Summarize the evidence from epidemiological studies on long-term exposure to air pollution on mortality and morbidity. Discipline—environmental epidemiology.
	(Rojas-Rueda et al., 2016) *Health impacts of active transportation in Europe* [33]	Study modelling the health impacts of using two active transport scenarios across five European cities. Main discipline—epidemiology. Europe.
**Cluster 2 (Blue)—Describing the fields**		
Papers describing the field of HIA, Health in EIA, and EIA with most papers making recommendations to strengthen fields. Mix disciplinary perspectives covering public health/medicine, environmental epidemiology, and social research with a tendency more towards positivist paradigm perspectives than cluster 3.		
	(Winkler et al., 2013) *Untapped potential of health impact assessment* [34]	Opinion piece describing the field of HIA. Makes recommendations for HIA to be demonstrated to be useful and beneficial. Discipline—environmental epidemiology. International.
	(Harris-Roxas et al., 2012) *Health impact assessment: the state of the art* [35]	Paper describing the state of the field of HIA. Discipline—public health—social research. International.
	(Bhatia and Wernham, 2008) *Integrating human health into environmental impact assessment: an unrealized opportunity for environmental health and justice* [36]	Reviews the role of HIA in EIA. Reviews case studies, statures, guidelines. Identifies supportive factors. Makes recommendations on how to strengthen integration of health into EIA. Discipline—medical, public health. United States.
	(Kemm, 2013) *Past achievement, current understanding, and future progress in Health Impact Assessment* [13]	Overview of field of HIA. Makes recommendations for future. Discipline—medicine, public health.
	(Morgan, 2012) *Environmental Impact Assessment: the state of the art* [37]	Paper describing the state of the field of EIA Reviews origins and development of field, current issues in relation to theory, practice and effectiveness, strengths, and weaknesses Discipline—geography International.
**Cluster 3 (Green)—Defining HIA**		
Cluster 3 focussed on defining and describing HIA. It included two main types of papers—1. large scale evaluations of HIA effectiveness; 2. papers defining and describing HIA. Overarching disciplinary perspective public health with a tendency towards disciplines with an implicit interpretivist paradigm perspective.		
	(Wismar and European Observatory on Health Systems and Policies, 2007) *The effectiveness of health impact assessment: scope and limitations of supporting decision-making in Europe* [38]	Reports findings of large-scale EU funded project to investigating the use and perceived effectiveness of HIA across Europe. Case studies—describing practice. Framework for understanding effectiveness. Discipline—political science. Europe.
	(WHO Regional Office for Europe, 1999) *Gothenburg Consensus Paper* [20]	Seminal paper defining and describing HIA. Discipline—NA/health policy. Europe.
	(Harris-Roxas and Harris, 2011) *Differing forms, differing purposes: A typology of health impact assessment* [5]	Describes a typology of HIA. Discipline—public health—social research. Australasia.
	(Bourcier, 2015) *An Evaluation of Health Impact Assessments in the United States, 2011—2014* [39]	Large scale evaluation project of HIAs. Case studies—describing practice. Focus on effectiveness. Discipline—public health, biology. North America.
	(Haigh et al., 2015) *What makes health impact assessments successful? Factors contributing to effectiveness in Australia and New Zealand* [40]	Large scale evaluation project. Focus on effectiveness—factors determining effectiveness. Discipline—public health, law. Australasia.
**Cluster 4 (Yellow) Quantitative Modelling Tools for HIA**		
Papers either describing quantitative health impact assessment tools or application. Main disciplinary perspective environmental epidemiology with a strong positivist paradigm perspective.		
	(Briggs, 2008) *A framework for integrated environmental health impact assessment of systemic risks* [41]	Findings from EU projects. Reviews current risk assessment approaches. Presents a framework for integrated environmental HIA. Discipline—geography, environmental epidemiology. Europe.
	(Veerman et al., 2005) *Quantitative health impact assessment: current practice and future directions* [42]	Reviews methods used in quantitative HIA. Identify areas for future research and development. Discipline—medicine, environmental epidemiology. International.
	(Lhachimi et al., 2012) *DYNAMO-HIA–A dynamic modelling tool for generic health impact assessments* [43]	Paper describing a quantitative modelling tool that can be used within HIA. Discipline—epidemiology. Europe.
	(Boshuizen et al., 2012) *The DYNAMO-HIA Model: an efficient implementation of a risk factor/chronic disease Markov Model for use in health impact assessment (HIA)* [44]	Paper also describing the use of the Dynamo HIA tool. Discipline—epidemiology, statistics. Europe.
	(Kulik et al., 2012) *Comparison of tobacco control scenarios: quantifying estimates of long-term health impact using the DYNAMO-HIA modelling tool* [45]	Paper describing application of the Dynamo HIA tool. Discipline—social epidemiology.
